# Endothelial-specific RIPK1 K376 mutation triggers kinase hyperactivation and embryonic lethality

**DOI:** 10.1016/j.gendis.2026.102258

**Published:** 2026-05-27

**Authors:** Zhuyang Wu, Lingxia Wang, Xiaoxia Wu, Ming Li, Haibing Zhang

**Affiliations:** CAS Key Laboratory of Nutrition, Metabolism and Food Safety, Shanghai Institute of Nutrition and Health, University of Chinese Academy of Sciences, Chinese Academy of Sciences, Shanghai 200031, China

Receptor-interacting protein kinase 1 (RIPK1) is a central integrator of survival and death signals, with its function tightly controlled by post-translational modifications.^1^ Ubiquitination at lysine 376 (K376), predominantly through K63-linked polyubiquitination, has been shown to prevent both apoptosis and necroptosis *in vitro* and *in vivo*.^2^ By generating RIPK1 K376R knock-in mice, which abolish ubiquitination at this site, we and others have demonstrated that *Ripk1*^*K376R/K376R*^ embryos invariably undergo lethality at embryonic day 12.5 (E12.5).^3^^,^^4^ More recently, by introducing a kinase-dead D138N mutation into the K376R background, we generated *Ripk1*^*K376R*,*D138N/K376R*,*D138N*^ mice and found that they survive to adulthood, thereby rescuing the embryonic lethality observed in *Ripk1*^*K376R/K376R*^ animals. These findings suggest that ubiquitination at K376 preserves normal embryonic development by restraining RIPK1 kinase activation-dependent cell death.

Since aberrant RIPK1 activation is a hallmark of various inflammatory and neurodegenerative disorders, a key unresolved question is which specific cell types require RIPK1 K376 ubiquitination during development to prevent embryonic lethality. Given prior evidence that endothelial cell death plays a critical role in embryonic vascular development, we generated endothelial cell–specific knock-in mutants (*Ripk1 K376R*^*fl/fl*^*Tie2-Cre*^*+*^) to investigate how RIPK1 activation triggered by loss of K376 ubiquitination in endothelial cells contributes to embryonic lethality.

Firstly, we generated endothelial cell-specific knock-in mice (*Ripk1 K376R*^*fl/fl*^*Tie2-Cre*^*+*^) by crossing *Ripk1 K376R*^*fl/+*^*Tie2-Cre*^*+*^animals with *Ripk1 K376R*^*fl/fl*^ mice via Cre-loxP–mediated recombination (Fig. S1A). Notably, no *Ripk1 K376R*^*fl/fl*^*Tie2-Cre*^*+*^pups were obtained at birth, suggesting embryonic lethality of the mutants. To determine the stage at which lethality occurs, embryos from the same mating strategy were collected at sequential developmental time points (Fig. 1A). At E11.5, *Ripk1 K376R*^*fl/fl*^*Tie2-Cre*^*+*^embryos and their yolk sacs appeared grossly normal. However, by E12.5, although the overall morphology of *Ripk1 K376R*^*fl/fl*^*Tie2-Cre*^*+*^embryos was indistinguishable from that of controls, stereomicroscopic analysis revealed systemic hemorrhage, growth retardation, and cardiac developmental delay (Fig. 1B; Fig. S1B). Histopathological examination further revealed hepatic blood island disintegration and vascular rarefaction with coagulative necrosis. Semi-quantitative scoring confirmed a severe and significant increase in both hemorrhage and vascular rarefaction scores in *Ripk1 K376R*^*fl/fl*^*Tie2-Cre*^*+*^embryos compared to controls (Fig. 1C; Fig. S3). Further analysis by terminal deox-ynucleotidyl transferase dUTP nick-end labeling (TUNEL) staining showed that *Ripk1 K376R*^*fl/fl*^*Tie2-Cre*^*+*^embryos had more TUNEL-positive cells than *Ripk1 K376R*^*fl/fl*^ embryos at E12.5 (Fig. 1D). Furthermore, quantitative reverse transcription polymerase chain reaction (qRT-PCR) analysis of E12.5 mutant embryos and yolk sac revealed a pronounced inflammatory signature, with significant upregulation of interleukin-6 (IL-6), tumor necrosis factor-alpha (TNF-α), C-X-C motif chemokine ligand 1 (CXCL1) and C-X-C motif chemokine ligand 2 (CXCL2) compared to controls (Fig. 1E; Fig. S1C). Consistently, immunofluorescence staining showed significantly increased cleaved caspase-3 (CC3) and phosphorylated RIPK3 (p-RIPK3) signals in mutant embryos compared with controls (Fig. 1F and G). Moreover, three-dimensional reconstruction of yolk sac vasculature precisely co-localized CC3^+^ signals with platelet endothelial cell adhesion molecule-1 (PECAM1^+^) cells (Fig. S1D). By E13.5 and E14.5, *Ripk1 K376R*^*fl/fl*^*Tie2-Cre*^*+*^embryos displayed severe anemia and overt developmental abnormalities, ultimately resulting in embryonic demise. Together, these findings demonstrate that the endothelial cell-specific RIPK1 K376R mutation causes mid-gestational lethality at E12.5.

**Figure 1:**
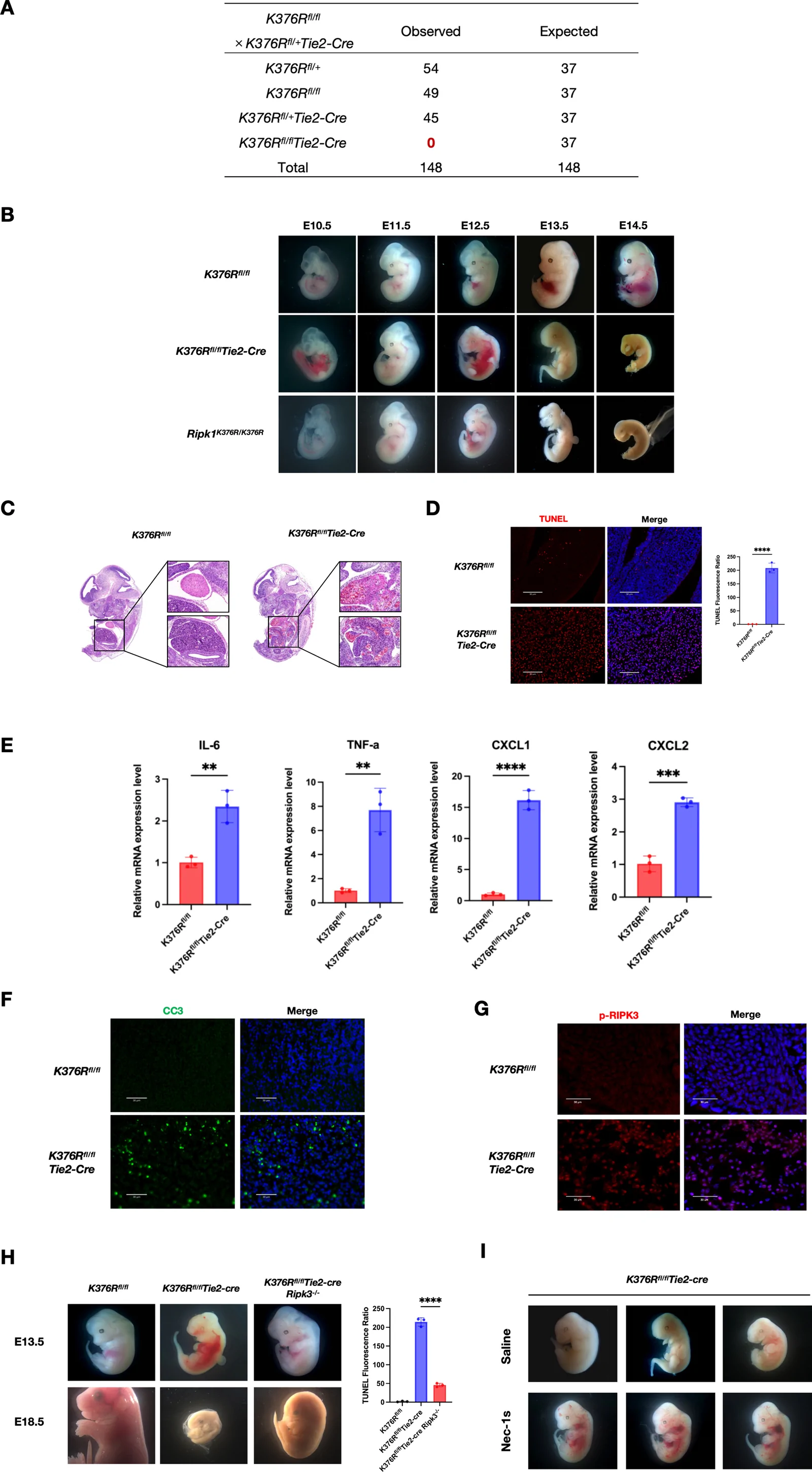
Endothelial RIPK1 K376R mutation induces embryonic lethality. **(A)** Observed numbers of embryos or live born pups of the indicated genotypes at various developmental stages from *K376R^fl^^/+^Tie2-Cre* and *K376R*^*fl/fl*^ mouse intercrosses. **(B)** Whole-mount dark field images of embryos with the indicated genotypes. Images are representative of embryos from E10.5 (*n* = 3), E11.5 (*n* = 3), E12.5 (*n* = 6), E13.5 (*n* = 7), and E14.5 (*n* = 4). **(C)** Representative hematoxylin and eosin (H&E)-stained mouse embryos of the indicated genotypes. Images are representative of *K376R*^*fl/fl*^ (*n* = 3) and *K376R*^*fl/fl*^*Tie2-Cre* mice (*n* = 3). **(D)** Representative TUNEL staining from mouse embryos of the indicated genotypes and quantitative cell death analysis. Images are representative of *K376R*^*fl/fl*^ (*n* = 3) and *K376R*^*fl/fl*^*Tie2-Cre* (*n* = 3) mice. **(E)** qRT-PCR analysis of inflammatory cytokine and chemokine expression in embryo homogenates at E12.5. Data are mean ± SEM. (*K376R*^*fl/fl*^ embryo: *n* = 3; *K376R*^*fl/fl*^*Tie2-Cre* embryo: *n* = 3). Statistical significance was determined using a two-tailed unpaired *t* test, ∗∗*p* < 0.01; ∗∗∗*p* < 0.001, ∗∗∗∗*p* < 0.0001. **(F)** Immunostaining for C–C-3 (green) from *K376R*^*fl/fl*^ and *K376R*^*fl/fl*^*Tie2-Cre* embryos. Images are representative of embryos from E12.5. **(G)** Immunostaining for p-Ripk3 (red) from *K376R*^*fl/fl*^ and *K376R*^*fl/fl*^*Tie2-Cre* embryos. Images are representative of embryos from E12.5. **(H)** Whole-mount dark field images of *K376R*^*fl/fl*^*Tie2-Cre^+^ Ripk3^−/−^* embryos from E13.5 and E18.5. Statistical results of TUNEL staining in liver sections in placentas of *K376R*^*fl/fl*^*Tie2-Cre^+^Ripk3^−/−^* at E13.5. **(I)** Pregnant *K376R*^*fl/fl*^*Tie2-Cre* females were fed with Nec-1s (50 mg/kg) or saline solution once per day since day 9.5 of gestation and sacrificed on day 13.5. Representative embryos with the indicated genotypes were presented.

Previous studies have shown that endothelial-specific deletion of *Fadd* or *Casp8* results in embryonic lethality due to RIPK3-dependent necroptosis, which is partially restored by *Ripk3* deficiency.^5^ To determine whether RIPK1 K376R-induced embryonic lethality occurred through a similar mechanism, we generated *Ripk1 K376R*^*fl/fl*^*Tie2-Cre*^*+*^*Ripk3*^*−/−*^ embryos. Remarkably, these double mutants developed normally at E13.5 with intact vascular networks and well-organized organ structures (Fig. 1H).

Consistent with these results, pharmacological inhibition of RIPK1 kinase activity with Necrostatin-1s (Nec-1s; 50 mg/kg daily from E9.5) partially restored embryonic viability (Fig. 1I; Fig. S1E). Moreover, given our previous findings that either combined deletion of *Fadd* and *Ripk3* or genetic inactivation of RIPK1 kinase activity can partially restore *Ripk1*^*K376R/K376R*^ mice and survival into adulthood,^3^ these results demonstrate that endothelial cell-specific RIPK1 K376R mutation promotes aberrant RIPK1 kinase activation, which drives embryonic lethality through synergistic induction of apoptosis and necroptosis. Although our data indicate that RIPK1 kinase activity is deregulated, as supported by functional rescue with Nec-1s and by downstream markers including p-RIPK3 and CC3, direct assessment of RIPK1 activation (e.g., p-RIPK1) was not performed, which represents a limitation of the study. Although genetic ablation of *Ripk3* or pharmacological inhibition of necroptosis partially improves embryonic survival, embryos ultimately fail to survive to birth, indicating that necroptosis alone is insufficient to account for the lethality. These observations support a model in which both necroptosis and apoptosis contribute to endothelial-specific RIPK1 K376R-induced embryonic lethality. However, the present study does not fully resolve the relative contributions of these two cell death pathways. Future studies employing combined inhibition of necroptosis and apoptosis, for example, through the use of *Ripk3*^*−/−*^, *Casp8*^*−/−*^ or *Fadd*^*−/−*^ endothelial-specific models, will be required to more precisely delineate the contribution of each mechanism.

RIPK1 is a critical regulator of cell death and inflammatory signaling, and its dysregulation has been implicated in multiple developmental and pathological contexts. Our findings identify endothelial cells as the critical origin of RIPK1 hyperactivation-driven embryonic lethality. Loss of K376 ubiquitination in endothelial cells unleashes RIPK1 kinase activity, inducing both apoptosis and necroptosis and resulting in vascular failure and embryonic lethality. In this study, we employed the Tie2-Cre driver to target the endothelial lineage, which is widely used and supported by our observation of extensive co-localization between the mutant phenotype (CC3/p-RIPK3) and the pan-endothelial marker PECAM1 (Fig. S1D). However, it has been reported that Tie2-Cre can also be active in subsets of hematopoietic and progenitor populations. Therefore, while our data strongly support a predominant endothelial contribution to the observed embryonic phenotype, a minor involvement of non-endothelial lineages cannot be formally excluded. Future studies employing hematopoietic-restricted Cre drivers, such as Vav-Cre, will be instrumental in definitively determining whether hematopoietic cells contribute to the defects in embryonic development observed in this model. Notably, two recent cases of autoinflammatory disease were associated with recessive compound heterozygous mutations in human RIPK1 (K377E/R390G), presenting with fever, lymphadenopathy, and skin rash. This cross-species conservation underscores the pathogenic relevance of RIPK1 dysregulation at this critical residue. Furthermore, the identification of endothelial RIPK1 ubiquitination as a key checkpoint provides new insight into the maintenance of vascular homeostasis and suggests that targeted modulation of RIPK1 kinase activity could represent a therapeutic strategy for vascular inflammatory diseases. Future work should determine whether pharmacological interventions can selectively restore this protective checkpoint in disease contexts.

## CRediT authorship contribution statement

**Zhuyang Wu:** Writing – original draft, Investigation, Formal analysis, Data curation, Conceptualization. **Lingxia Wang:** Supervision, Resources. **Xiaoxia Wu:** Validation. **Ming Li:** Visualization, Project administration. **Haibing Zhang:** Writing – review & editing, Funding acquisition.

## Ethics declaration

All research procedures were approved by Shanghai Institute of Nutrition and Health, Chinese Academy of Sciences.

## Funding

This work was supported by the 10.13039/501100018537National Science and Technology Major Project for Chronic Non-communicable Diseases (No. 2024ZD0531300), the 10.13039/501100012166National Key Research and Development Program of China (No. 2022YFA0807300), the Shanghai Postdoctoral Excellence Program (No. 2024713, 2024715) and the Shanghai Municipal Science and Technology Major Project.

## Conflict of interests

The authors declare no competing interests.
