# Supplementary material for: Endothelial-specific RIPK1 K376 mutation triggers kinase hyperactivation and embryonic lethality
Source: Genes Dis. 2026 May 27;14(2):102258. doi: 10.1016/j.gendis.2026.102258 (PMC13631518; doi:10.1016/j.gendis.2026.102258)
Supplement: Supplementary file 1 [file mmc1.docx]

**Figure S1. Endothelial RIPK1 K376R mutation induces embryonic lethality.**

**A** Schematic overview of strategy to generate *Ripk1 K376R^fl/fl^Tie2-Cre^+^* mice.The mutation was confirmed by sequencing.

**B** Supplemetary whole-mount dark field images of embryos with indicated genotypes from E12.5.

**C** qRT-PCR analysis of inflammatory cytokines and chemokines expression in yolk sacs homogenates at E12.5. Data are mean ± s.e.m. (*K376R^fl/fl^* embryo: n = 3; *K376R^fl/fl^Tie2-Cre* embryo: n = 3). Statistical significance was determined using a two-tailed unpaired t test, **P < 0.01; ***P < 0.001, ****P < 0.0001.

**D** Immunostaining for PECAM (green) and C-C-3(red) on yolk sacs from *K376R^fl/fl^* and *K376R^fl/fl^Tie2-Cre* embryos. Images are representative of yolk sacs from E12.5.

**E** Pregnant *K376R^fl/fl^Tie2-Cre* females were fed with Nec-1s (50 mg/kg) or saline solution once per day since day 9.5 of gestation and sacrificed on day 13.5.

**Figure S2. Model for endothelial RIPK1 K376 mutation-induced embryonic lethality.**

**A** The gain-of-function K376 mutation in RIPK1 causes kinase activation, promoting downstream apoptosis and necroptosis. This leads to endothelial cell death and consequent embryonic lethality in mice.

**Figure S3. Semi-quantitative scoring analysis of the hemorrhage and vascular rarefaction phenotypes observed from E12.5 and E13.5 embryos.**

**A** Semi-quantitative scoring analysis of the hemorrhage phenotypes observed from E12.5 embryos.

0=No hemorrhage observed. 1=Focal, minor hemorrhage in one tissue. 2=Multifocal hemorrhage in one or two tissues. 3=Widespread, severe hemorrhage in multiple tissues (e.g., liver, brain, subcutaneous region).

**B** Semi-quantitative scoring analysis of the vascular rarefaction phenotypes observed from E13.5 embryos.

0=Normal, dense vascular network. 1=Mild reduction of vascular structures. 2=Moderate reduction with clear areas of loss. 3=Severe disruption with only scattered vessels remaining.

**Detailed materials & methods：**

**Mice.** All mice were housed in a specific pathogen-free (SPF) facility at Shanghai Institute of Nutrition and Health, Chinese Academy of Sciences.All newly constructed mouse lines were backcrossed onto the C57BL/6 background for more than eight generations. Animal experiments were conducted in accordance with the guidelines of the Institutional Animal Care and Use Committee of the Shanghai Institute of Nutrition and Health, University of Chinese Academy of Sciences, Chinese Academy of Sciences.

**Immunostaining.** Briefly, tissues were collected in ice-cold 1xPBS and then fixed in

PFA (4%) at 4 °C overnight. After washing in cold PBS for three times, tissues were

embedded in the OCT (Sakura) and stored at -80°C. Cryosections 10 µm in thickness

were collected on positively charged slides and stored at -40°C. For immunostaining,

tissue sections were blocked with blocking buffer (0.1% Triton X-100/5% BSA/PBS)

for 1 hour at room temperature, followed by antibody staining overnight. The next day, tissue sections were washed three times and incubated with antibody (Alexa fluorescence-conjugated) for 1 hour at room temperature, and then washed with PBS

for three times and mounted with mounting medium (containing DAPI).

**RT-PCR.**Total RNAs were extracted using Trizol (9108, Takara) from mice embryos. Transcript levels of indicated genes were quantified by quantitative RT-PCR on an ABI 7500 real-time PCR instrument with SYBR Green. Relative expression of gene transcripts was analysed by using the 2^−ΔΔCt^ method.

**Statistical analysis.** Data presented in this article are representative results of at least

three independent experiments. A log-rank (Mantel-Cox) test was used to compare

mouse survival. The statistical significance of data was evaluated by Student’s t test,

one-way-ANOVA and two-way ANOVA. The statistical calculations were performed

with GraphPad Prism software.
